# Gender Comparison of Psychological Comorbidities in Tinnitus Patients – Results of a Cross-Sectional Study

**DOI:** 10.3389/fnins.2020.00704

**Published:** 2020-07-07

**Authors:** Alessandra Fioretti, Eleonora Natalini, David Riedl, Roland Moschen, Alberto Eibenstein

**Affiliations:** ^1^Tinnitus Center, European Hospital, Rome, Italy; ^2^University Clinic of Medical Psychology, Medical University of Innsbruck, Innsbruck, Austria; ^3^Department of Biotechnological and Applied Clinical Sciences, University of L’Aquila, L’Aquila, Italy

**Keywords:** tinnitus, gender, metacognition, depression, anxiety, tinnitus distress

## Abstract

**Background:**

In the last decades, research focused on gender-related features in patients with tinnitus has often led to controversial results. The complex clinical picture of tinnitus patients often consists of an interdependent relationship between audiological symptoms and co-occurrent psychological disorders, which can complicate the diagnostic evaluation.

**Methods:**

Therefore, we studied 107 patients with tinnitus, investigating their psychological comorbidities in the light of gender differences. All patients were evaluated with ENT/audiological and psychological examination to consider presence/absence, type and gender distribution of psychopathological comorbidities. Patients completed questionnaires on tinnitus distress (Tinnitus Handicap Inventory, THI), anxiety (Beck Anxiety Inventory, BAI), depression (Beck Depression Inventory, BDI), metacognition (Metacognition Questionnaire-30, MCQ-30) and worry (Penn State Worry Questionnaire). The influence of gender on the relationship between tinnitus distress and psychological comorbidities was investigated with simple moderation analyses using the SPSS PROCESS macro.

**Results:**

The total sample included 65 male and 42 female patients (60.7 vs. 39.3%), matched for age and duration of tinnitus. We found no significant differences for tinnitus distress (THI total score, THI subscales) and MCQ-30 subscales, except for the control over thoughts, where men showed significantly higher scores than women (*p* = 0.045). Also, in our sample women showed significantly higher values for depression (BDI total score, *p* = 0.019), anxiety (BAI total score, *p* = 0.010) and worries (PSQW total score, *p* = 0.015). Moderation analyses revealed a significant influence of gender on the relationship of tinnitus distress with depression: higher scores of tinnitus distress were associated with significantly elevated levels of depression amongst men. No further gender influences could be observed in our sample.

**Discussion:**

In conclusion, our results indicate general gender differences for psychological comorbidities in tinnitus patients, with women reporting more depression, anxiety and worries. Men, on the other hand, showed a higher need to control their thoughts. Additionally, our results indicate that men might have more coping problems with increasing levels of tinnitus distress, leading to increased depressive symptoms. Nevertheless, several gender related aspects in tinnitus patients remain unclear, thus warranting the need future studies in this field.

## Introduction

Tinnitus is a multifactorial disorder and it involves the perception of sounds, such as ringing or buzzing in the ear or head without detectable external source (Cima et al., 2019). Recent studies reveal that about 30% of adults can experience tinnitus but overall prevalence varied over 8-fold from 5.1 to 42.7% (McCormack et al., 2016). Based on a survey conducted in 2014 on 2,952 individuals, Gallus et al. (2015) found that any tinnitus was reported by 6.2% of Italian adults and severe tinnitus by 1.2%. Moreover, approximately one-third of adults with tinnitus experience it as bothersome and feel impaired in their daily performance (Tunkel et al., 2014). As reported by Cima et al. (2019), bothersome tinnitus might be better described as a negative emotional and auditory experience. Tinnitus can be further distinguished into acute (<3 months), sub-acute (3–6 months) or chronic (>6 months) (Cima et al., 2019).

Etiology of tinnitus seems to be very heterogeneous, though in many cases it occurs after cochlear damages following noise trauma, age, ototoxic drugs, inflammatory diseases or hearing loss. Studies focused on the relation between gender and tinnitus distress often show conflicting results (Meric et al., 1998; Erlandsson and Holgers, 2001; Pinto et al., 2010; Seydel et al., 2013). Psychological comorbidities may either be pre-existent or induced by tinnitus. Core symptoms of tinnitus often mask psychiatric and psychological comorbid symptoms. Apart from phenotypical sex differences, tinnitus patients may also show gender differences in regard to coexisting psychopathologies. Thus, several studies focusing on gender differences in psychological comorbidities among tinnitus patients have been conducted.

Gomaa et al. (2014) reported gender differences for depression, anxiety, and stress among patients with tinnitus. However, they did not conduct sex specific analyses of the association between tinnitus severity and psychiatric distress. Caldirola et al. (2016) investigated the role of worry in 54 patients (29 males, 25 females) with chronic tinnitus and sensorineural hearing loss. Tinnitus-related anxiety, depression symptoms and handicap were significantly associated with the tendency to worry, but they found no associations between tinnitus distress and gender. Udupi et al. (2013) reported similar results in 50 patients (31 males, 19 females) with tinnitus, with tinnitus severity being significantly correlated with depressive symptoms as well as state and trait anxiety. Other factors – including age, gender or hearing status – did not significantly influence tinnitus severity.

Other authors reported gender differences in tinnitus related suffering. In a cross-sectional study, Han et al. (2019) evaluated 134 female and 114 male patients with tinnitus using the THI, BDI, the Korean version of Brief Encounter Psychosocial Instrument (BEPSI-K), and various characteristics of the patients’ tinnitus (including loudness, awareness, annoyance, and effect on life). While the authors found no significant gender difference in tinnitus severity, men showed a stronger association between tinnitus severity and depressive symptoms than women. Additionally, a significant association between stress and tinnitus severity was only found for male participants. Another study assessed gender differences in Positron emission tomography-computed tomography (PET-CT) results in tinnitus patients (Shlamkovich et al., 2016). In their study, 60% of the patients showed an increased uptake in the upper temporal gyrus (UTG) on either side. In their sample, men showed a significant increased uptake in the UTG, both in the subsample of patients with unilateral tinnitus as well as in the total sample.

Previous research has also underlined the presence of higher anxiety and depression levels and suicide attempts in female tinnitus patients. Bashir et al. (2017) found higher scores of depression and anxiety among female patients with tinnitus. While Gallus et al. (2015) found no overall sex differences regarding the prevalence of (chronic) tinnitus, severe levels of tinnitus distress were more frequent in women. Lugo et al. (2019) reported in a cross -sectional study a sex-dependent association of tinnitus with suicide attempts, with severe tinnitus associated with suicide attempts in women but not in men. Furthermore, women reported more tinnitus complaints then men.

To summarize, the evidence base for sex differences in tinnitus comorbidities is still inconclusive. Thus, the aim of the present study was to investigate sex specific patterns of psychological comorbidities, including measure for meta-cognitions, anxiety, depression and worry. Results are analyzed for clinically relevant confounders such as age and tinnitus duration.

## Materials and Methods

### Sample and Setting

A sample of *n* = 107 outpatients evaluated at the Tinnitus Center of European Hospital (Rome) between April 2018 and April 2019 was included in this cross-sectional study. To be included in the study, patients (a) had tinnitus, (b) were older than 18 years, (c) spoke Italian fluently, and (d) had no apparent cognitive impairment or major psychiatric/neurological disorders (schizophrenia, Alzheimer’s disease, Parkinson’s disease). The patients were evaluated by an ENT specialist and a psychologist, and they completed the questionnaires as part of the routine clinical practice. Written informed consent was obtained by all patients.

### Measures

#### Tinnitus Sample Case History (TSCH)

The Italian version of the TSCH was used to assess sociodemographic and clinical data. The TSCH was developed by the Tinnitus Research Initiative to facilitate standardized assessment of sociodemographic and clinical data in tinnitus research (Langguth et al., 2007). The questionnaire consists of 35 items on background (i.e., age, gender), tinnitus history (i.e., loudness, pitch, perception at onset, tinnitus location, percentage of awake time aware of tinnitus) and related conditions (i.e., hearing impairment, noise annoyance, vertigo/dizziness, headache, TMJ disorders).

#### Tinnitus Handicap Inventory (THI)

The THI (Newman et al., 1996) is a widely used self-report questionnaire in tinnitus research, allowing the assessment of the impact of tinnitus in daily life. It consists of 25 items which can be summarized to a total score as well as a functional, emotional, and catastrophic subscale. Based on the total score tinnitus severity can be graded from slight (grade 1) to catastrophic (grade 5). Good reliability (α = 0.94) and validity was reported for the total score of the Italian THI version (Passi et al., 2008). A THI score <36 was considered to indicate a compensated tinnitus, while a score > 36 was considered to indicate a decompensated tinnitus (Salviati et al., 2013; Altissimi et al., 2016).

#### Beck Anxiety Inventory (BAI)

The BAI (Beck et al., 1988) is a self-administered tool to assess the severity of anxiety over the last 7 days. It consists of 21-items which can be rated on a 4-point scale ranging from 0 (“not at all”) to 3 (“severely”). Based on the total score, anxiety can be classified as minimal (0–7), mild (8–15), moderate (16–25), and severe (26–63).

#### Beck Depression Inventory (BDI)

The BDI-II (Beck et al., 1996) is a self-report instrument to assess depressive symptoms over the previous 2 weeks, consisting of 21 items. Based on the total score, depression can be classified as minimal (0–13), mild (14–19), moderate (20–28) or severe (29–63).

#### Metacognition Questionnaire-30 (MCQ-30)

The MCQ-30 (Wells and Cartwright-Hatton, 2004) is a 30-item questionnaire with 5 subscales: (1) positive beliefs about worry (pos); (2) negative beliefs about the controllability of thoughts and danger of worry (neg); (3) cognitive confidence (CC); (4) beliefs about the need to control thoughts (NC); and (5) cognitive self-consciousness (CSC). Higher scores on the total score as well as the subscales indicate higher levels of dysfunctional metacognitions.

#### Penn State Worry Questionnaire (PSWQ)

The PSWQ (Meyer et al., 1990) is a 16-item self-administered scale designed to measure worry. The items are scored on a 5-point Likert-type scale (1-Not at all typical of me to 5-Very typical of me). Based on the total score, the patients’ worries can be classified as low (16–39), moderate (40–59) and high (60–80).

#### Audiometry

Pure tone audiometry was carried out into an audiological cabin using a clinical audiometer (Madsen Itera II, GN Otometrics). Normal hearing was defined by threshold <25 dB HL in all frequencies tested between 250 and 8.000 Hz.

### Statistical Analyses

Descriptive statistics are presented for men and women separately and group differences were analyzed using independent sample *t*-tests and χ^2^-tests. Associations between tinnitus distress and the assessed psychological variables were analyzed with Pearson correlation coefficients and gender differences between all psychological variables using independent sample *t*-tests. Duration of tinnitus was dichotomized into acute (i.e., <3 months) and sub-acute/chronic tinnitus (>3 months) due to the group sizes. To evaluate the influence of multiple physical comorbidities, the mean number of reported comorbidities (i.e., hearing problems; hyperacusis; headache; vertigo/dizziness; TMD; neck pain; other pain) was correlated with the mean THI, BDI and BAI scores. *P*-values < 0.05 (two-sided) were considered statistically significant.

**FIGURE 1:**
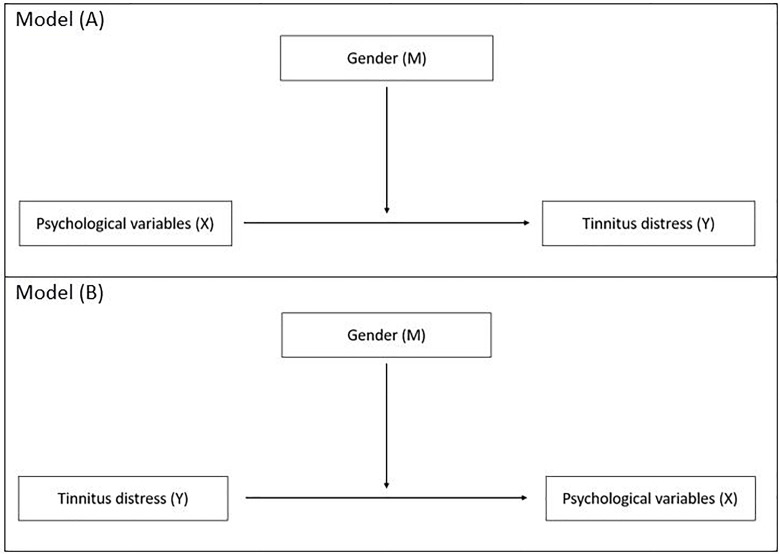
Moderator analyses of gender (moderator M) on the relationship of the psychological variables (i.e., depression, anxiety, worries, need for control) with tinnitus distress.

To investigate the potential influence of sex on the relationship of the psychological variables (i.e., MCQ-subscales, BDI total score, BAI total score, PSWQ total score) with tinnitus distress, two moderator analyses were calculated: in model (A) psychological variables were entered as independent variables, sex as moderator variable and tinnitus distress as dependent variable; in model (B) the direction of the association was changed, i.e., tinnitus distress was entered as independent variable, sex as moderator variable and psychological variables as dependent variables. The models are visualized in Figure 1.

Psychological variables were entered in the models if (a) there were significant sex differences for the variable and (b) they were significantly associated with tinnitus distress. Moderator analyses were calculated using the SPSS PROCESS macro (v3.4) (Hayes, 2013). All moderation analyses were completed utilizing a bootstrapping procedure with 5000 bootstrapped samples. Significance of the indirect effect is determined by examining the 95% confidence interval (CI) of the sampling distribution of the mean. Confidence intervals that do not include zero are considered statistically significant at the 0.05 level. Statistical analyses were performed with IBM SPSS (v22.0).

## Results

A total of *n* = 107 patients was included in the analyses. The mean age was 49.1 (SD: 13.9) years and 60.7% of the sample was male. The majority of patients (75.7%) reported to suffer from their tinnitus for longer than 6 month and most patients (73.8%) described their tinnitus as continuously present. Details on patient characteristics can be found in Table 1. Overall, 19.6% of the patients reported a very mild tinnitus severity (grade I), while 28.0% reported mild (grade II), 29.0% moderate (grade III), 15.9% severe (grade IV), and 7.5% very severe tinnitus distress (grade V). Thus, about half of the patients reported a compensated tinnitus (47.7%), while the other half reported a decompensated tinnitus (52.3%). Based on the pure tone audiometry (250–8.000 Hz), 68 patients had a hearing loss. No gender difference was found regarding hearing loss (*p* = 0.82).

**TABLE 1:** Sociodemographic and clinical data.

	Men (*n* = 65)		Women (*n* = 42)			
	*Mean*	*(SD)*	*Mean*	*(SD)*	*t-value*	*p-value*
Age	50.2	(13.2)	47.3	14.9	*1.05*	*0.29*
Tinnitus loudness (0–100)	50.6	(23.1)	54.8	(27.2)	*0.76*	*0.45*
Awareness of tinnitus ^a^	81.2	(28.1)	77.5	(28.0)	*0.59*	*0.56*
Annoyed by tinnitus ^a^	51.0	(34.4)	47.8	(30.1)	*0.44*	*.66*
	*n*	*%*	*n*	*%*	*χ^2^*	*p-value*
Tinnitus: family history	15	24.6%	16	38.1%	2.42	0.12
*Missing*	*4*	*6.2%*	*1*	*2.4%*		
Duration tinnitus						
<3 months	13	20.0%	4	9.5%	2.63	0.27
3–6 months	5	7.7%	2	4.8%		
>6 months	46	70.8%	35	83.3%		
*Missing*	*1*	*1.5%*	*1*	*2.4%*		
Tinnitus perception at onset						
Gradual	**48**	**73.8%**	**23**	**54.8%**	**4.15**	**0.04**
Abrupt	**8**	**12.3%**	**11**	**26.2%**		
Missing	9	13.8%	1	2.4%		
Pulsating tinnitus	10	15.4%	8	19.0%	0.27	0.61
*Missing*	*1*	*1.5%*	*1*	*2.4%*		
Tinnitus location						
right ear	9	13.8%	6	14.3%	1.31	0.73
Left ear	16	24.6%	7	16.7%		
Both ears	35	53.8%	27	64.3%		
Inside the head	4	6.2%	2	4.8%		
*Missing*	*1*	*1.5%*	*–*	*–*		
Tinnitus loudness varies from day to day	42	64.6%	29	69.0%	0.30	0.59
*Missing*	*1*	*1.5%*	*1*	*2.4%*		
Tinnitus manifestation					0.01	0.92
Intermittent	14	21.5%	9	21.4%		
Constant	49	75.4%	30	71.4%		
*Missing*	*2*	*3.1%*	*3*	*7.1%*		
Tinnitus sound					**8.49**	**0.037**
Tone	**36**	**55.4%**	**13**	**31.0%**		
Noise	**12**	**18.5%**	**12**	**28.6%**		
Crickets	**7**	**10.8%**	**3**	**7.1%**		
Other	**7**	**10.8%**	**11**	**26.2%**		
*Missing*	*3*	*4.6%*	*3*	*7.1%*		
Objective hearing problem ^b^	42	68.9%	26	66.7%	0.05	0.82
*Missing*	*4*	*6.2%*	*3*	*7.1%*		
Subjective hearing problem	24	36.9%	15	35.7%	0.01	0.97
*Missing*	*2*	*3.1%*	*3*	*7.1%*		
Hyperacusis	**15**	**23.1%**	**19**	**45.2%**	**10.45**	**0.005**
*Missing*	*4*	*6.2%*	*2*	*4.8%*		
Headache	**17**	**26.2%**	**23**	**54.8%**	**9.24**	**0.002**
*Missing*	*1*	*1.5%*	*1*	*2.4%*		
Vertigo/dizziness	**10**	**15.4%**	**15**	**35.7%**	**5.84**	**0.016**
*Missing*	*2*	*3.1%*	*1*	*2.4%*		
Temporomandibular disorder	18	27.7%	16	38.1%	1.94	0.16
*Missing*	*2*	*3.1%*	*4*	*9.5%*		
Neck pain	33	50.8%	27	64.3%	3.40	0.07
*Missing*	*0*	*0.0%*	*3*	*7.1%*		
Other pain	13	20.0%	14	33.3%	2.68	0.10
*Missing*	*5*	*7.7%*	*4*	*9.5%*		

*^*a*^ Percentage of awake time; ^*b*^ pure tone audiometry (250–8.000 Hz). Statistically significant clinical data (*P*-values < 0.05) are reported in bold.*

The mean number of comorbidities was significantly associated with the THI (*r* = 0.21, *p* = 0.034), BDI (*r* = 0.20, *p* = 0.039) and BAI total score (*r* = 0.23, *p* = 0.016). Most patients reported at least one physical comorbidity, with *n* = 12 (11.2%) having no comorbidities, *n* = 69 (64.5%) 1–3 comorbidities, and *n* = 26 (24.3%) four or more comorbidities. The mean number of comorbidities was significantly higher for women than for men (*t* = 3.1, *p* = 0.003).

No gender differences were found for tinnitus loudness, duration of tinnitus or tinnitus location, and most other tinnitus characteristics. Yet, women significantly more often reported an abrupt tinnitus onset significantly than men (*p* = 0.04) and showed significant differences in the sound profile of their tinnitus. There also was a significantly higher prevalence of hyperacusis (*p* = 0.005), headache (*p* = 0.002) and vertigo/dizziness (*p* = 0.016) in women than in men. Patients with headache reported hyperacusis (54.1 vs. 21.0%; *p* = 0.001) and temporomandibular disorders (51.4 vs. 22.2%; *p* = 0.003) significantly more often than patients without headache. As for vertigo/dizziness, patients with headache reported a higher prevalence than patients without headache, yet this difference was not statistically significant (33.3 vs. 18.5%; *p* = 0.086).

As previously reported (Maas et al., 2017; Lopez-Escamez and Amanat, 2020), a higher heritability was found for bilateral tinnitus in men at any age and younger women (<40 years). While young women with a bilateral tinnitus (*n* = 9) reported a notable higher percentage of tinnitus complaints in their familial history than men (*n* = 33) with bilateral tinnitus (55.6 vs. 24.2%), this difference was not statistically significant (*p* = 0.072).

Patients with acute tinnitus showed no significant differences regarding tinnitus distress (*p* = 0.49), nor with depression (*p* = 0.22), anxiety (*p* = 0.12), or worries (*p* = 0.49) when compared to patients with sub-acute or chronic tinnitus. Yet, both negative beliefs about uncontrollability and danger of worry (*p* = 0.003) and cognitive self-consciousness (*p* = 0.030) were significantly higher in patients with acute tinnitus than in patients with sub-acute or chronic tinnitus. No further significant differences were found for the other MCQ subscales.

### The Association of Tinnitus Distress With Meta-Cognitions, Anxiety, Depression and Worry

In our sample higher levels of tinnitus distress were significantly associated more depression, anxiety and worries. Regarding metacognitions, higher tinnitus distress was associated with negative beliefs about the controllability of thought, as well as with a higher need to control thoughts. No association was found between tinnitus distress and positive beliefs about worry, cognitive confidence or cognitive self-consciousness. For details see Table 2.

**TABLE 2:** Correlations of tinnitus distress and meta-cognitions, anxiety, depression, and worries.

	MCQ pos	MCQ neg	MCQ CC	MCQ NC	MCQ CSC	PSWQ	BDI	BAI
THI total score	0.13	0.38***	0.15	0.32**	0.10	0.45***	0.68***	0.47***
MCQ pos		0.26**	0.26**	0.34***	0.39***	0.11	−0.02	0.02
MCQ neg			0.11	0.49***	0.43***	0.61***	0.51***	0.50***
MCQ CC				0.09	0.09	0.20*	0.23*	0.33**
MCQ NC					0.47***	0.31**	0.29**	0.26**
MCQ CSC						0.36***	0.19	0.15
PSWQ total score							0.63***	0.64**
BDI								0.68**

*p < 0.05, **p < 0.01, ***p < 0.001; THI, Tinnitus Handicap Inventory; MCQ, Metacognition Questionnaire; pos, positive beliefs about worry; neg, negative beliefs about the controllability of thoughts and danger of worry; CC, cognitive confidence; NC, beliefs about the need to control thoughts; CSC, cognitive self-consciousness; PSWQ, Penn State Worry Questionnaire; BDI, Beck Depression Inventory; BAI, Beck Anxiety Inventory.

### Gender Differences in Tinnitus Distress, Meta-Cognitions, Depression, Anxiety and Worries

To investigate gender differences for the assessed psychological variables a set of independent sample *t*-tests was calculated. While no gender effect was found for tinnitus distress, women reported significantly higher scores for anxiety, depression and worries. Regarding meta-cognitions, men reported significantly stronger beliefs about the need to control thoughts than women. For details see Table 3.

**TABLE 3:** Gender differences of THI, MCQ, PSWQ, BDI, and BAI.

	Men		women			
	Mean	(SD)	Mean	(SD)	*t*-value	*p*-value
THI total score	39.0	(25.4)	43.7	(21.8)	0.99	0.32
THI functioning subscale	17.8	(12.0)	19.9	(10.6)	0.91	0.36
THI emotional subscale	12.6	(9.2)	14.4	(8.7)	0.99	0.32
THI catastrophic subscale	8.6	(5.5)	9.5	(5.0)	0.87	0.39
MCQ pos	10.8	(4.2)	9.7	(3.2)	1.42	0.16
MCQ neg	13.0	(4.1)	13.2	(4.3)	0.26	0.80
MCQ CC	10.9	(4.2)	10.9	(4.8)	0.02	0.98
**MCQ NC**	**11.3**	**(3.5)**	**9.9**	**(3.1)**	**2.03**	**0.045**
MCQ CSC	15.4	(4.0)	15.0	(3.7)	0.44	0.66
**PSWQ**	**45.2**	**(11.3)**	**51.0**	**(12.5)**	**2.47**	**0.015**
**BDI total**	**9.1**	**(6.6)**	**12.5**	**(8.3)**	**2.40**	**0.019**
**BAI total**	**8.1**	**(7.4)**	**12.6**	**(10.2)**	**2.62**	**0.010**

*SD, standard deviation; THI, Tinnitus Handicap Inventory; MCQ, Metacognition Questionnaire; pos, positive beliefs about worry; neg, negative beliefs about the controllability of thoughts and danger of worry; CC, cognitive confidence; NC, beliefs about the need to control thoughts; CSC, cognitive self-consciousness; PSWQ, Penn State Worry Questionnaire; BDI, Beck Depression Inventory; BAI, Beck Anxiety Inventory; statistically significant values printed bold.*

### The Moderating Effect of Gender on the Relationship of Tinnitus Distress and Depression, Anxiety, Worries and Need for Control (Meta-Cognition)

In the first model of the moderation analyses, the potential gender influence on the influence of the included psychological variables with the extent of tinnitus distress were tested. While there was a significant association of all psychological variables with tinnitus distress (*p* < 0.001), no statistically significant moderating effect of gender could be observed (*p* > 0.05).

**FIGURE 2:**
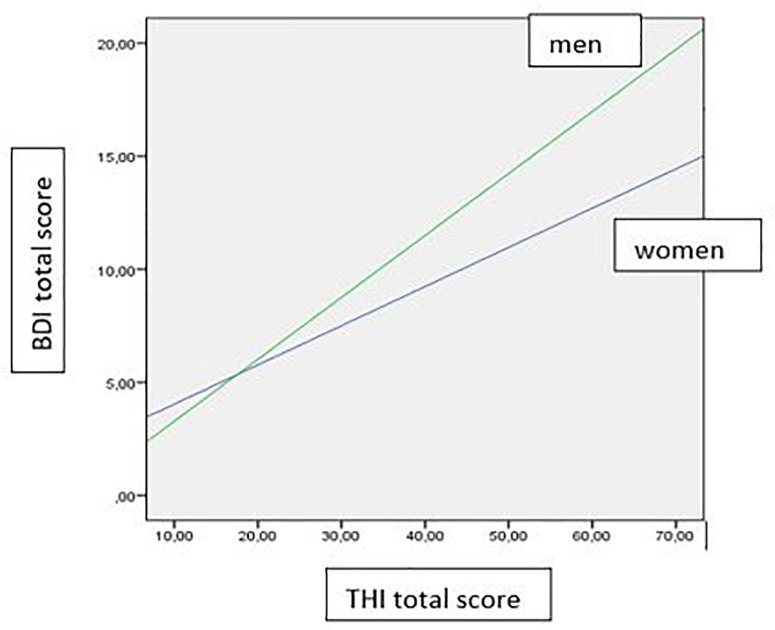
Gender effect on the influence of the THI total score on the BDI total score.

In the second moderation model the reversed effect was tested, thus investigating the moderation effect of gender on the influence of tinnitus distress on the psychological variables. Similar to model (A), there was a significant influence of tinnitus distress on all included psychological variables (*p* < 0.001). Additionally, gender significantly moderated the influence of tinnitus distress on depression (*p* = 0.03): while lower levels of tinnitus distress were associated with lower levels depression in men than in women. This effect was reversed with increasing levels of tinnitus distress as shown in Figure 2. For details see Table 4 and Figure 2.

**TABLE 4:** Results of the moderator analyses using the PROCESS macro.

			coeff	se	*t*	*p*	LLCI	ULCI
**Model (A)**	**THI** *R*^2^ = 0.15, *MSE* = 508.76 *p* = 0.010	MCQ NC	3.12	0.80	3.91	<0.001	1.54	4.70
		Sex	27.80	15.00	1.87	0.07	−1.77	57.73
		MCQ NC × Sex	−1.92	1.38	−1.39	0.17	−4.65	0.81
	**THI** *R*^2^ = 0.21, *MSE* = 471.96 *p* < 0.001	**PSWQ**	**1.05**	**0.24**	**4.36**	**<0.001**	**0.57**	**1.52**
		Sex	16.13	18.06	0.89	0.37	−19.69	51.95
		PSWQ × Sex	−0.34	0.36	−0.95	0.35	−1.06	0.38
	**THI** *R*^2^ = 0.47, *MSE* = 313.96 *p* < 0.001	**BDI**	**2.56**	**0.34**	**7.63**	**<0.001**	**1.90**	**3.23**
		Sex	4.34	6.23	0.70	0.49	−8.02	16.71
		BDI × Sex	−0.68	0.47	−1.43	0.16	−1.61	0.26
	**THI** *R*^2^ = 0.25, *MSE* = 448.43 *p* < 0.001	**BAI**	**1.77**	**0.36**	**4.98**	**<0.001**	**1.06**	**2.48**
		Sex	7.78	6.52	1.19	0.24	−5.16	20.73
		BAI × Sex	−0.87	0.48	−1.81	0.07	−1.82	0.09
**Model (B)**	**MCQ NC** *R*^2^ = 0.17, *MSE* = 10.07 *p* < 0.001	**THI**	**0.06**	**0.02**	**3.87**	**<0.001**	**.03**	**.09**
		Sex	−0.08	1.32	0.06	.95	−2.70	2.54
		THI × Sex	−0.04.	0.03	1.30	.19	−.09	.02
	**PSWQ** *R*^2^ = 0.24, *MSE* = 114.80 *p* < 0.001	**THI**	**0.21**	**0.05**	**3.94**	**<0.001**	**0.10**	**0.31**
		Sex	3.73	4.47	0.84	0.41	−5.13	12.60
		THI × Sex	0.02	0.09	0.26	0.80	−0.16	0.21
	**BDI** *R*^2^ = 0.51, *MSE* = 28,36 *p* < 0.001	**THI**	**0.17**	**0.03**	**6.61**	**<0.001**	**0.12**	**0.23**
		Sex	−1.76	2.22	0.79	0.43	−6.17	2.64
		**THI** × **Sex**	**0.10**	**0.05**	**2.18**	**.03**	**0.01**	**0.19**
	**BAI** *R*^2^ = 0.27, *MSE* = 59.24 *p* < 0.001	**THI**	**0.15**	**0.04**	**4.01**	**<0.001**	**0.08**	**0.23**
		Sex	1.80	3.21	0.56	0.58	−4.56	8.17
		THI × Sex	0.05	0.07	0.67	0.50	−0.09	0.18

*Coeff, coefficient; se, standard error; LLCI and ULCI, lower and upper levels for confidence interval; MSE, mean squared error; statistically significant values printed bold.*

## Discussion

The purpose of this study was to investigate gender differences in tinnitus distress and associated psychological comorbidities in patients with tinnitus.

About a quarter of the patients in our sample reported severe or very severe tinnitus distress. In accordance with previous research, increased levels of tinnitus distress were associated with higher levels of depression, anxiety and worries (Durai and Searchfield, 2016; Pattyn et al., 2016; Salazar et al., 2019). Furthermore, we found that higher tinnitus distress was also associated with positive beliefs about worry, negative beliefs about the controllability of thoughts and danger of worry, cognitive confidence, beliefs about the need to control thoughts, and cognitive self-consciousness.

In line with the literature, our results showed that tinnitus was accompanied by hearing loss in 68% of the patients. Similar risk factors responsible for cochlear impairment may be an explanation for this link (Shargorodsky et al., 2010; Martines et al., 2015). In our sample there was no statistically significant gender difference in tinnitus distress, neither in the THI total scale nor in any of the subscales, which is in accordance with previous studies (Han et al., 2019). Yet, there was a distinct gender effect for the assessed psychological comorbidities, with women reporting higher anxiety, depression and worries than men. These gender effects are consistent with previous studies, who also found higher anxiety and depression (Gallus et al., 2015; Pattyn et al., 2016; Bashir et al., 2017; Strumila et al., 2017; Ziai et al., 2017), as well as higher level of distress (Seydel et al., 2013) and increased likelihood for suicide attempts (Lugo et al., 2019) in women.

Comorbidities as hypertension, insomnia and migraine have been previously associated with chronic tinnitus. The role of arterial hypertension as a risk factor for tinnitus is well known from the literature because blood pressure alterations could affect the cochlear microcirculation, yet no gender differences were reported (Figueiredo et al., 2015; Martines et al., 2015; Yang et al., 2015; Figueiredo et al., 2016). Recently, some authors also reported an association between masked hypertension, arterial stiffness and tinnitus (Gun et al., 2019; Gedikli et al., 2020). Additionally, sleep disorders and insomnia are frequently associated with tinnitus (Fioretti et al., 2013; Aazh et al., 2019). As a consequence, the insomnia Cognitive Behavioral Therapy (i-CBT) was proposed as a treatment to reduce insomnia and distressing tinnitus (Marks et al., 2019). Chen et al. (2019) found that patients with non-migraine headache are at significantly greater risk of tinnitus than those without chronic headache. Patients with migraine have also an increased risk to develop cochlear disorders and in particular tinnitus (Hwang et al., 2018). Langguth et al. (2017) described that the laterality and severity of primary headache and tinnitus are significantly related. The co-occurrence of tinnitus and other comorbidities like vertigo, hyperacusis, headache and depression could suggest the presence of a somatoform disorder. In line with the current literature, we found a significantly higher prevalence of headache (*p* = 0.002) in women than in men. Thus, our findings support the idea to explore different causes of tinnitus to classify multiple subtypes and to find different treatments for patients with tinnitus (Landgrebe et al., 2010; McFerran et al., 2019).

In our sample, there was also a higher prevalence of hyperacusis, headache and vertigo/dizziness in women than in men. This may not be a tinnitus specific finding, since previous studies have found an increased likelihood for hyperacusis (Paulin et al., 2016), headache (Buse et al., 2013) and vertigo or dizziness (Kurre et al., 2012) in women. As proposed by other authors, these findings are supported by the hypothesis that in women homeostasis of labyrinthic fluids may be altered by hormone alterations in the menstrual cycle, pregnancy and menopause causing balance and hearing disorders (Ishii et al., 2009; Reiss and Reiss, 2014). Yet, in our study there was no increased prevalence of temporomandibular disorders, which is in contradiction to previously described prevalence rates (Vielsmeier et al., 2012; Algieri et al., 2017; Edvall et al., 2019). Data from literature reports a greater heritability for bilateral tinnitus in men and young women (Maas et al., 2017; Lopez-Escamez and Amanat, 2020). In our sample, there was a notably higher prevalence of a familial history of tinnitus in younger women (<40 years) than in men. Yet, this difference was not statistically significant. Apart from the psychological comorbidities and clinical features, there was also a gender effect regarding the patients meta cognitions, which–to our knowledge – has not been investigated in patients with tinnitus so far. The concept of meta cognitions describes how people reflect their own cognitive processes. In our sample, men reported significantly stronger beliefs about the need to control thoughts than women. So far, no studies have described a general gender difference regarding metacognitions (e.g., Quattropani et al., 2014). Future studies could confirm our results giving the opportunity to offer tailored therapy, like Metacognition Therapy (Wells, 2009), to treat the worry and the need to control thoughts.

The mediation analyses showed no significant interaction of gender on the relationship of the psychological comorbidities or meta-cognitions on tinnitus distress. Yet, there was an inversed relationship: while in our sample men had lower depression scores than women, we also found that with increasing tinnitus distress, the level of depression increased significantly stronger in men than in women. This partially reproduced the findings of Han et al. (2019), who found that tinnitus severity did not significantly differ between the gender groups but partial correlations between tinnitus severity and depressive symptoms were stronger in males than in females. One explanation for our results could be, that men might have more problems to cope with higher levels of tinnitus distress and thus may react more depressed. Since this is a cross-sectional study, of course we cannot make any causal assumptions, but our results emphasize the importance of psychological comorbidities as predictors of tinnitus severity in clinical management.

This study has some ***limits***. First, the included sample is comparably small and multicenter studies with larger sample would contribute to clarify the results found in our study. Second, concerns have been raised on the reliance on self-reported symptoms like temporomandibular disorders, sleep disorders, headache and hyperacusis extrapolated from the answers of the TSCH. Nevertheless, the TSCH is a validated instrument for standardized collection of information about the characteristics of the tinnitus patient and by the time of the data collection its use still was recommended to facilitate comparability of studies in tinnitus research (Langguth et al., 2007). Third, we did not investigate presence of clinical comorbidities (hypertension, migraine, insomnia), the level of hearing loss, tinnitus pitch and loudness or the role of traits of personality among tinnitus patients as this was not the focus of the study. We focused our attention on the psychological comorbidities, and we only analyzed the presence of normal hearing/hearing loss measured with a standard pure tone audiogram (250–8,000 Hz). No absolute correlation between hearing loss and tinnitus was demonstrated since, as recently proposed, a mild hearing impairment could be present in patients with tinnitus and yet be missed by a standard pure tone audiogram (PTA) measured at octave or half octave intervals from 250 to 8,000 Hz (Xiong et al., 2019). Thus, a strong audiological evaluation would require a high definition audiogram with the study of high frequencies (Liberman et al., 2016; Lefeuvre et al., 2019), DPOAE (Xiong et al., 2019) and the threshold equalizing noise (TEN) test between 500 and 4 kHz (Kara et al., 2020) to detect hidden hearing loss and synaptopathy. Also, as proposed by Simoes et al. (2019), neuroticism and extraversion might be relevant markers of tinnitus distress over time and may be used to statistically distinguish patient groups with clinically relevant changes of tinnitus distress. Finally, we did not assess the duration of the psychiatric comorbidities, thus it is not entirely clear, whether patients suffered from pre-existing psychiatric complaints or if these symptoms unfolded associated with the tinnitus onset.

## Conclusion

In conclusion, our results indicate general gender differences for psychological comorbidities in tinnitus patients, with women reporting more depression, anxiety and worries. Men, on the other hand, showed a higher need to control their thoughts. The question about the distinction between female and male tinnitus patients remains open, considering that (1) both genetic and psychosocial risk factors may contribute to this gender difference, (2) epidemiological studies reports higher prevalence of anxiety disorders and depression in female than in man [World Health Organization (WHO), 2017]. Despite the high heterogeneity of prevalence estimates, there is emerging evidence of substantial prevalence of anxiety disorders generally (3.8–25%), and particularly in women (5.2–8.7%) (Remes et al., 2016). Women have higher rates of major depression compared to men and, in average, the ratio is 2:1 (Bromet et al., 2011). Yet, there is substantial evidence that the gender difference in major depression diagnoses and depression symptoms peaks in adolescence and the gender gap then narrows and remains stable in adulthood (Salk et al., 2017). It could be hypothesized that gender differences in tinnitus and its relief arise from an interaction of genetic, anatomical, physiological, neuronal, hormonal, psychological and social factors which modulate tinnitus differently in the sexes. Our results suggest that recognition of the evidence underlying sex differences in tinnitus will guide development of treatments and provide better options for patients that are tailored to their physical and psychological comorbidities.

## Data Availability Statement

The datasets for this article are not publicly available since the consent of the patients to do so was not obtained. Requests to access the datasets should be directed to the corresponding author DR.

## Ethics Statement

The studies involving human participants were reviewed and approved by Ethics committee of the University of L’Aquila ID number 18/2020. The patients/participants provided their written informed consent to participate in this study.

## Author Contributions

AF: conceptualization, methodology, supervision, writing (original draft), and project administration. EN: writing (original draft) and data curation. DR: methodology, formal analysis, and writing (original draft). RM: writing (review and editing). AE: writing (review and editing) and supervision. All authors contributed to the article and approved the submitted version.

## Conflict of Interest

The authors declare that the research was conducted in the absence of any commercial or financial relationships that could be construed as a potential conflict of interest. The handling Editor declared a past co-authorship with one of the authors AF.
